# Label-free plasma proteomics identifies haptoglobin-related protein as candidate marker of idiopathic pulmonary fibrosis and dysregulation of complement and oxidative pathways

**DOI:** 10.1038/s41598-020-64759-x

**Published:** 2020-05-08

**Authors:** Mayank Saraswat, Sakari Joenväärä, Tiialotta Tohmola, Eva Sutinen, Ville Vartiainen, Katri Koli, Marjukka Myllärniemi, Risto Renkonen

**Affiliations:** 1 Transplantation Laboratory, Faculty of Medicine, University of Helsinki, Helsinki, Finland; 2 HUSLAB, Helsinki University Hospital, Helsinki, Finland; 3 Department of Pulmonary Medicine, Heart and Lung Center, Helsinki University Hospital, Helsinki, Finland; 4 Individualized Drug Therapy Research Program, Faculty of Medicine, University of Helsinki, Helsinki, Finland; 5 Present Address: Department of Laboratory Medicine and Pathology, Mayo Clinic, Rochester, MN 55905 USA; 6 Present Address: Center for Molecular Medicine, National Institute of Mental Health and Neurosciences (NIMHANS), Bangalore, 560029 India; 7 Present Address: Manipal Academy of Higher Education (MAHE), Manipal, 576104 Karnataka India

**Keywords:** Mass spectrometry, Diagnostic markers

## Abstract

Idiopathic pulmonary fibrosis (IPF) is a lung parenchymal disease of unknown cause usually occurring in older adults. It is a chronic and progressive condition with poor prognosis and diagnosis is largely clinical. Currently, there exist few biomarkers that can predict patient outcome or response to therapies. Together with lack of markers, the need for novel markers for the detection and monitoring of IPF, is paramount. We have performed label-free plasma proteomics of thirty six individuals, 17 of which had confirmed IPF. Proteomics data was analyzed by volcano plot, hierarchical clustering, Partial-least square discriminant analysis (PLS-DA) and Ingenuity pathway analysis. Univariate and multivariate statistical analysis overlap identified haptoglobin-related protein as a possible marker of IPF when compared to control samples (Area under the curve 0.851, ROC-analysis). LXR/RXR activation and complement activation pathways were enriched in t-test significant proteins and oxidative regulators, complement proteins and protease inhibitors were enriched in PLS-DA significant proteins. Our pilot study points towards aberrations in complement activation and oxidative damage in IPF patients and provides haptoglobin-related protein as a new candidate biomarker of IPF.

## Introduction

Idiopathic pulmonary fibrosis is chronic, progressive, interstitial pneumonia of unknown cause usually occurring in older adults and presents with usual interstitial pneumonia (UIP) in histopathological and/or radiological findings. Current data suggests that the incidence of IPF has been increasing in some parts of the world including Europe^1^. The diagnosis of IPF is largely clinical and radiological and laboratory investigations are often not helpful although they can be used to rule out other conditions. The common symptoms include breathlessness on exertion, decreasing pulmonary function, bibasilar inspiratory crackles and finger clubbing in 50% of the patients^2–4^. Decline in respiratory function can be slow and progressive or rapid and accelerated giving rise to variable survival pattern. Damage in IPF is usually irreversible and unpredictable and prognosis is extremely poor^2–4^. According to collaborative efforts of the American Thoracic Society, the European Respiratory Society, the Japanese Respiratory Society, and the Latin American Thoracic Association, diagnosis requires exclusion of other known causes of interstitial lung disease (environmental exposure, drug toxicities and connective tissue disease), presence of a UIP pattern on high-resolution computed tomography (HRCT) and/or combination of UIP pattern in HRCT and surgical lung biopsies^2^.

IPF can lead to the death of patients in 3–5 years after onset of symptoms^2^. Options for therapy of IPF are controversial due to lack of knowledge of common suitable symptoms for initiating therapy and until a few years ago, lung transplant was the only option. Two antifibrotic agents have been approved by the FDA and EMA. There exists a lack of understanding of molecular mechanisms driving the disease as well as suitable detection and monitoring biomarkers. Larger efforts are needed to find suitable minimally invasive biomarkers of the IPF to help early diagnosis and therapy onset.

We have performed label-free plasma proteomics on 36 plasma samples including 17 confirmed IPF cases (2011 ATS/ERS/JRS/ALAT diagnostic guidelines^2^) and 19 healthy controls. The sample collection was done in accordance with 2011 ATS/ERS/JRS/ALAT guidelines. Since then, 2018 guidelines have become available^5^ however the major diagnostic criterion remains unchanged. We have quantified 167 proteins with 2 or more unique peptides out of which 74 were significantly different between the IPF and controls by t-test. FDR correction reduced this number to 66. Multivariate statistical analysis methods were employed to find suitable high-confidence biomarkers. Their performance was evaluated by ROC curve analysis.

## Results

### Metadata

Detailed patient characteristics (including measurements of lung function tests of IPF cases) for the study population are given in Supplementary Table 1 in Supplementary dataset. Nineteen healthy individuals (5 females, 14 males) and 17 IPF cases (3 females and 14 males) comprise the study population. The median age for the healthy group was 73 years and 71 years for IPF cases. The current study is designed according to a binary case-control comparison.

### Label-free Proteomics and differential proteins

Hundred and sixty six proteins were quantified with 2 or more unique peptides. Total peptides identified included 5416 out of which 4261 were unique to various proteins (Supplementary table 2 in Supplementary dataset). Confidence score ranged from 6.4 for carbonic anhydrase 1 to 3093 for complement C3. Levels of Seventy four proteins were significantly different between the groups (IPF vs controls, t-test p value <0.05, Supplementary table 2 in Supplementary dataset) out of which 10 had a highest mean in IPF and 64 had highest mean in controls. The median power to separate the groups was 0.908 among these proteins. Benjamini-Hochberg FDR correction was applied to the dataset and 66 proteins were differentially expressed between the groups (FDR corrected p value <0.05, Supplementary Table 2). Ten out of these 66 proteins had increased amounts in IPF patients and 56 others had increased amounts in controls.

### Further statistical analysis

**Figure 1:**
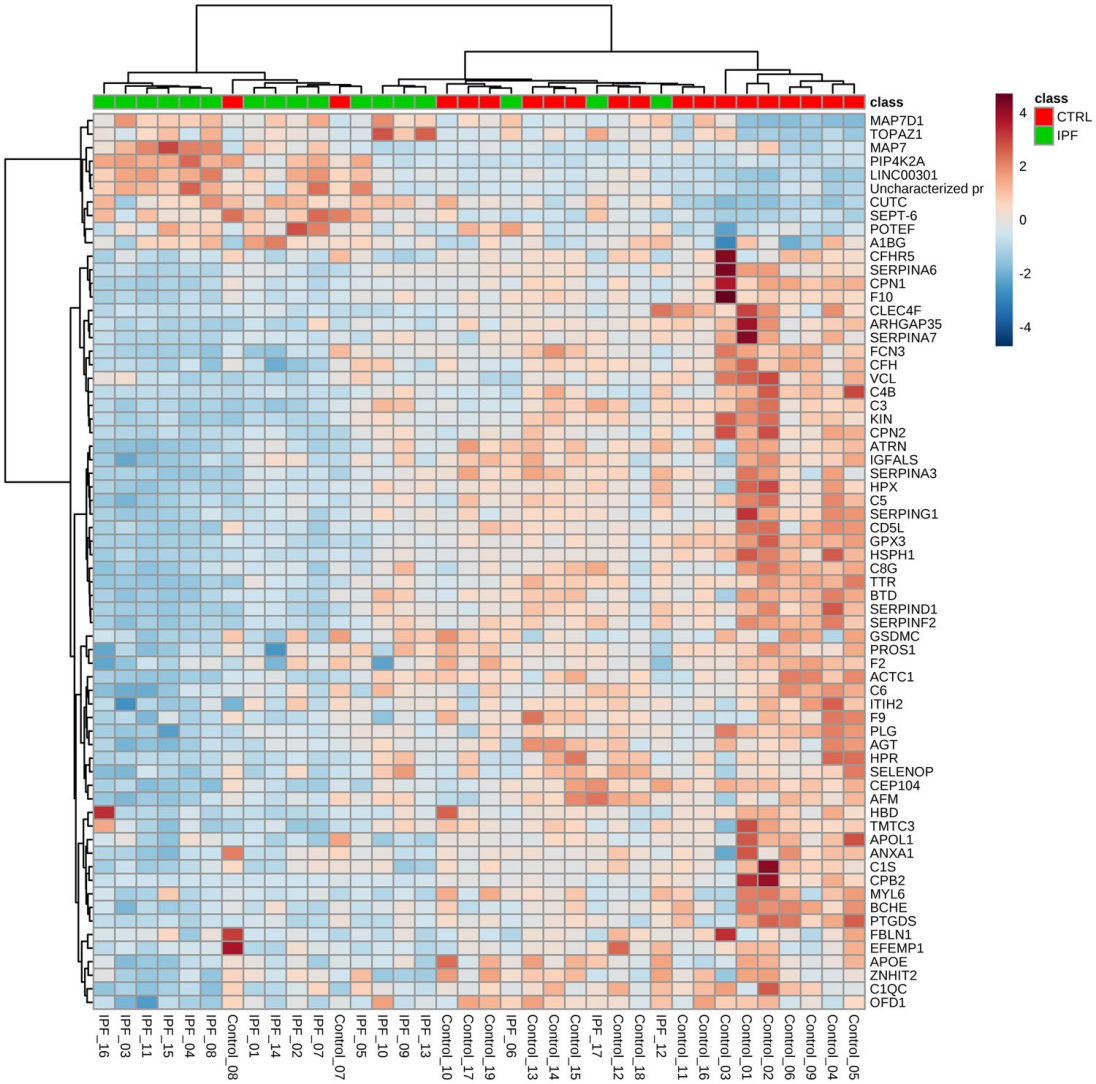
Hierarchical Clustering heatmap. Hierarchical clustering was performed for Controls and Idiopathic pulmonary fibrosis (IPF) comparison considering sixty two Benjamini-Hochberg FDR corrected t-test p value (<0.05) passing proteins. Metaboanalyst 4.0 was used for performing the clustering. Horizontal axis is all the samples analyzed in the study and vertical axis denotes Uniprot accessions for 66 proteins. On top of the heatmap are controls samples in green squares and IPF samples (Cases) in red squares. Dendrogram for samples is shown on top of the heatmap and proteins’ dendrogram on left side of the heatmap. Dark blue to dark red colour gradient denotes lower to higher expression.

Hierarchical clustering was performed for all proteins (Supplementary Figure 1) as well as differentially expressed proteins (FDR corrected p value <0.05, Fig. 1) to test if the groups can be separated by unsupervised statistical techniques. Clustering the groups by all proteins produced mixed groups (Supplementary Figure 1) which can be expected with majority of the proteins being non-differing between the groups. When significantly differing proteins were used for clustering the sample groups there was good separation between IPF and controls however few samples overlapped into each other’s group (Fig. 1). To tease out the natural contrast in protein expression between the groups, clustering was also performed by forcing the samples in each group to be organized together utilizing 66 differentially expressed proteins (Supplementary Figure 2). Here, 10 proteins increased in IPF patients and 56 in controls could be clearly seen to have a contrasting pattern.

**Figure 2:**
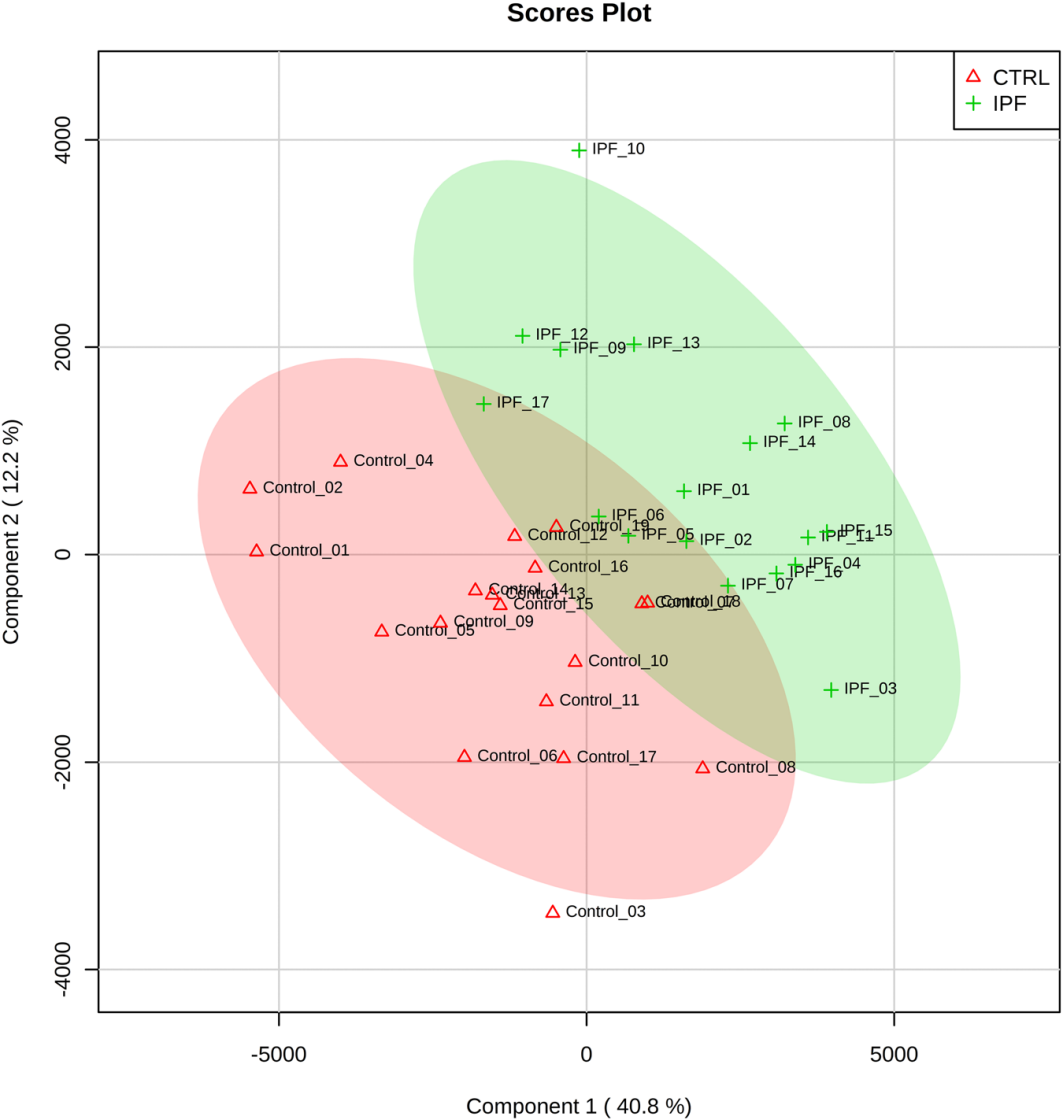
Scores plot for Partial least square-discriminant analysis. Score plot shows Idiopathic pulmonary fibrosis (IPF) in red and controls in green circles. A five component modeling was performed by PLS-DA using MetaboAnalyst 4.0 program. Ovals show 95% confidence interval for both IPF (red oval) and controls (green oval). This is a scatter plot for 2 components having the greatest variations. Observations that are similar will fall close to each other displaying a clustering-like pattern. Component 1 (X-axis) contains 40.8% of the total variation and component 2 contains 12.2%. Plotting the scores will display separation of the samples in a score plot, as shown.

Encouraged by clustering, we performed partial least square-discriminant analysis modeling on pareto-scaled data to find out the separation between IPF and controls and simultaneously identify the proteins whose expression can separate the 2 groups. All 166 proteins were used for PLS-DA modeling and scores plot was generated (Fig. 2). The scores plot showed separation between the two groups based on their expression (Fig. 2). However score plot cannot be used to gauge the separation of groups on face value because of the common problem of overfitting of the model^6^. Ten-fold cross validation was employed to test the fit (R^2^), accuracy and predictive ability (Q^2^) which can be seen in the Supplementary Figure 3. Ten fold CV produced reasonably good model parameters with very significant predictive accuracy (Q^2^) which was highest with top 4 components (latent variables).

### Selection of biomarkers

Candidate biomarker selection was carried out in two steps; coarse selection and fine selection. Coarse selection involved volcano plot and VIP values generated by PLS-DA modeling and fine selection was done by finding significant proteins which overlap between these 2 techniques. As a last step, Receiver operating characteristic curve analysis was employed to calculate the area under the curve values.

### Coarse selection

**Figure 3:**
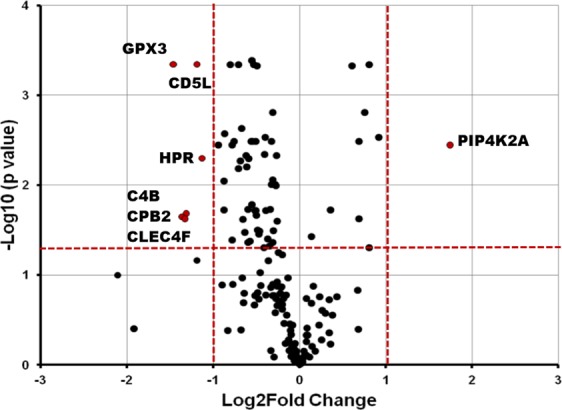
Volcano Plot. X-axis is the Log_2_ of linear fold change (IPF/Controls) and Y-axis is the negative Log_10_ of the Benjamini-Hochberg corrected t-test p value. Vertical red dashed lines denote a linear fold change of 2 in either direction (IPF/Controls or Controls/IPF). Horizontal red dashed line denotes a cutoff of 0.05 for the FDR corrected p value. Any protein which falls to the left or right of vertical red dashed lines and above horizontal red dashed line (p value <0.05) is deemed to be significant different between the groups.

For selecting biomarker candidates, a volcano plot was generated by putting negative logarithm of the FDR corrected p value on Y axis and Log2 fold change on X axis (Fig. 3). Proteins having FDR corrected p value of less than 0.05 (Horizontal red dashed line, Fig. 3) and fold change of at least 2 (2 Vertical dashed lines, Fig. 3) were selected in the first step of coarse selection. Seven proteins passing these criteria are described in Table 1.

**Table 1:** Volcano plot significant proteins are shown in the table. These proteins passed the cutoff of having less than 0.05 of FDR corrected t-test p value and having a linear fold change of at least two in either direction, either higher in Idiopathic pulmonary fibrosis (Case) or controls. Uniprot accession, protein name (Description), Benjamini-Hochberg false discovery rate corrected p-values, total peptide count, unique peptides unique to the given protein, confidence score of identification, fold Changes and highest mean conditions are given in the table.

Accession	Description	FDR corrected p-values	Peptide count	Unique peptides	Confidence score	Fold change	Power	Highest mean	Lowest mean
P00739	Haptoglobin-related protein	0.005030	27	5	365.7	2.19	0.990	CONTROL	CASE
P22352	Glutathione peroxidase 3	0.000454	8	6	60.7	2.76	0.999	CONTROL	CASE
O43866	CD5 antigen-like	0.000454	5	5	33.5	2.28	0.999	CONTROL	CASE
P48426	Phosphatidylinositol 5-phosphate 4-kinase type-2 alpha	0.003576	4	2	18.5	3.35	0.993	CASE	CONTROL
P0C0L5	Complement C4-B	0.020712	161	6	1863.9	2.48	0.857	CONTROL	CASE
Q96IY4	Carboxypeptidase B2	0.023661	6	3	43.3	2.51	0.995	CONTROL	CASE
Q8N1N0	C-type lectin domain family 4 member F	0.02245	3	2	21.5	2.58	0.974	CONTROL	CASE

Going back to the PLS-DA model, the most significant top protein features able to classify the 2 group of samples were selected based on variable influence on projections (VIP) values (Supplementary Table 2 in Supplementary dataset). Proteins having VIP value of 1 or higher were considered the best classifiers (Supplementary Table 2 in Supplementary dataset). Thirty proteins had a VIP value of 1 or more, but as a cross validation, only the proteins which were significant by FDR corrected p value proteins list and also having VIP value of more than 1 were considered leaving us with 24 proteins (Supplementary Table 2 in Supplementary dataset). These proteins are called “top significant in PLS-DA modeling” here onwards. Complement C3, Testis- and ovary-specific PAZ domain-containing protein 1 (TOPAZ1) and hemopexin among others came out as the most important features to classify the IPF vs controls groups.

### Fine selection

**Figure 4:**
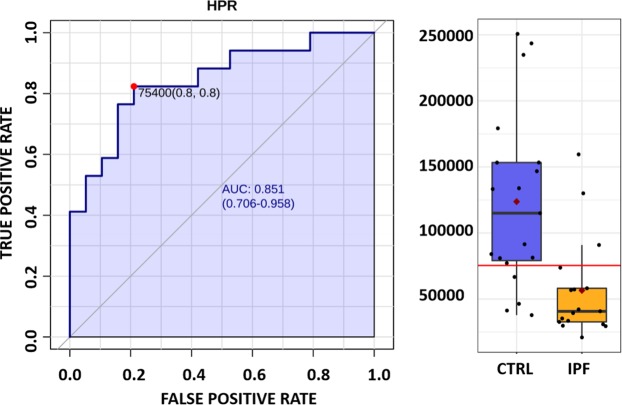
Receiver operating characteristics curve analysis of Haptoglobin-related protein to differentiate idiopathic pulmonary fibrosis (IPF) from controls. Sensitivity (Y-axis) is plotted against 1-specificity for haptoglobin-related protein (Uniprot Accession: P00739) to differentiate between IPF and controls. Area under the curve value is shown on the ROC curve together with 95% confidence interval. At cutoff of 75400 (normalized abundance level), 80% of sensitivity and 80% of specificity was achieved. At right side of ROC curve, a box plot is shown to compare the two groups with individual samples shown as empty circles for cases (IPF) and black filled circles for controls.

To finally select candidate biomarkers able to classify the IPF patients from healthy controls, proteins overlapping between the FDR significant proteins and PLS-DA VIP list were selected. An additional filter was applied and proteins were required to have a fold change of at least two. Comparing volcano plot significant proteins and proteins selected by VIP method gave us only one protein as a strong marker, Haptoglobin-related protein. This protein was reduced 2.2 times in IPF patients plasma compared to healthy controls. It was identified with 5 unique peptides with confidence score of 365.71 with power of 0.99 to separate the groups. As a last step, ROC curve analysis was performed and area under the curve (AUC) value of 0.851 was found (Fig. 4). At cutoff of normalized abundance value of 75400, it had 80% sensitivity and 80% selectivity.

### Pathway analysis

**Figure 5:**
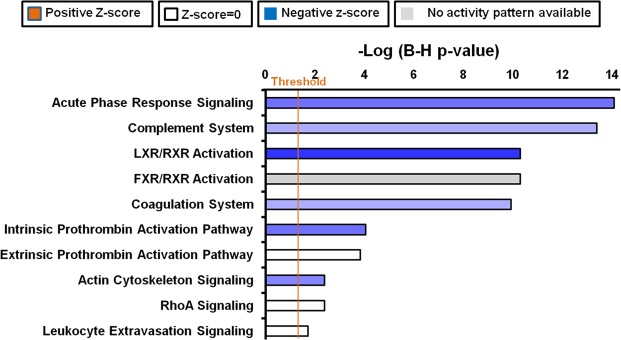
Canonical pathways. Bar chart showing significant canonical pathways (B-H p-value <0.05) generated by Ingenuity Pathway Analysis software “Core analysis” of significantly different proteins between Idiopathic pulmonary fibrosis and controls obtained using FDR corrected t-test (<0.05). “Data were analyzed through the use of IPA (QIAGEN Inc., https://www.qiagenbioinformatics.com/products/ingenuitypathway-analysis)”.

Ingenuity pathway analysis was used for performing “Core analysis” on IPF vs Controls proteomics dataset. It gave several enriched canonical pathways (B-H p-values <0.05) in the dataset (Fig. 5) including LXR/RXR/FXR activation, acute phase response signaling, complement system and coagulation system among others. Remember that in top proteins selected by VIP values in PLS-DA modeling there were several complement proteins and coagulation regulators (Supplementary Table 2 in Supplementary Dataset). These proteins were also significant by FDR corrected t-test p values in IPF vs Controls.

## Discussion

2011 diagnostic guidelines of ATS/ERS/JRS/ALAT require a largely clinical and radiological diagnosis of IPF. The median time for diagnosis of IPF from symptom onset is 12 months (data for North America) and a quarter of the patients are not diagnosed for up to 26 months^7^. This is unfortunate as the median survival time of patients with IPF ranges from 30–42 months^8,9^. Not many treatment options remain at advanced stage of the disease and access to lung transplantation within reasonable time varies according to geographical location among other factors. There is a very strong need for IPF detection and monitoring biomarkers to make further improvements in treatment of the disease. Additionally, identification of dysregulated systemic pathways will also help further the understanding of the disease and provide avenues for further research into diagnosis and treatment. We have performed label-free plasma proteomics in 37 individuals including 17 confirmed cases of IPF and 20 healthy controls with the aim of finding novel markers of the disease.

Hierarchical clustering provided good separation of the IPF vs control samples which only improved when differentially expressed proteins’ data was employed, as can be expected in case of true differential expression. Further, PLS-DA was performed after pareto scaling of the data. In OMICS data, variable usually have very different ranges which creates sub-optimal modeling and interpretation of such analysis is difficult. Variable are transformed by scaling to circumvent this problem. Pareto scaling is normally used in multivariate data analysis^10,11^ to upscale the medium features without increasing baseline noise unlike unit variance (UV) scaling. PLS-DA is sensitive to scaling and different features having impact on separation and the real separation itself can be more easily revealed on scaled data^10^. It is to be noted that large values receive more attention in pareto scaling but it is beneficial in analysis of proteomics data and increases confidence in results as larger peaks will have better signal/noise ratio compared to peaks close to background. In a clinical proteomics study, biomarker selection is often the goal and precision, predictive ability, sensitivity and selectivity are of obvious importance. In a binary comparison, there are a number of univariate and multivariate feature selection methods available to scientists. However, large systematic studies where these feature selection methods have been compared to each other in terms of their performance are lacking. Together with varying degree of statistical know-how in biomedical scientists, it leads to a situation where there are no consensus guidelines as to what method should be used in any given situation. It has been shown that when sample size reach n = 15 per group, t-test combined with FDR correction is the best performing (precision, true positives) method  compared to nearest shrunken centroid, support vector machine, principal component discriminant analysis and PLS-DA^12^. Of note, large within class variance and low sample size (n ≤ 6) leads to poor t-test performance in selecting the best discriminating features^12^. Same study found that PLS-DA is largely unaffected by low sample size or between- and within-class variability. Combining the strength of both of these methods, we employed coarse selection of biomarkers using FDR corrected t-test p values (additionally requiring FC ≥ 2) and PLS-DA significant proteins (VIP ≥ 1). In current study, PLS-DA provided  30 proteins with VIP values of more than one, 24 of which (Supplementary Table 2 in Supplementary dataset) were also significant by FDR corrected t-test p value (<0.05). A volcano plot was generated by plotting FDR corrected t-test p values and fold change. Seven significant proteins were found having fold change of more than two (Table 1). In the fine selection of biomarkers, proteins overlapping between the volcano plot significant and VIP ( ≥ 1) significant were proposed as candidate biomarkers. This provided HPR as the only biomarker candidate. Three pertinent themes are sequentially discussed in this section namely; Volcano plot significant proteins, PLS-DA significant proteins and pathways and HPR as proposed candidate biomarker to discriminate IPF from healthy individuals.

Volcano Plot significant proteins included Phosphatidylinositol 5-phosphate 4-kinase type-2 alpha (PIP4K2A), which was 3.35 fold increased in plasma of IPF patients. There are no reports linking PIP4K2A to IPF or fibrosis in general however, lipid species phosphatidylinositol 5-phosphate acts as a second messenger and elicits innate antiviral response^13^. PIP4K2A converts Phosphatidylinositol 5-phosphate to phosphatidylinositol 4,5-bisphosphate regulating its levels which might lead to diminished innate immunity. In line with this hypothesis, sustained induction of innate immunity is known to amplify fibrosis by several mechanisms^14^. Six other volcano plot significant proteins had reduced levels in IPF patients’ plasma including Complement C4B, Glutathione peroxidase 3 (GPx3), Haptoglobin related protein (HPR), CD5 antigen-like (CD5L) and Carboxypeptidase B2 (CPB2). Of note, several proteases in our dataset had significantly reduced expression in IPF plasma including Carboxypeptidase N. Glutathione peroxidase 1 (GPx1) works downstream of Nrf2/Bach1, a major intracellular antioxidant effector axis. It has been shown that perfenidone treatment inhibits Bach1 and improves GPx1 levels in bleomycin-induced pulmonary fibrosis^15^. GPx3 is a secreted antioxidant plasma counterpart of GPx1 and GPx2. It works in detoxification of reactive oxygen species by glutathione. We found 2.76 times reduced levels of GPx3 in IPF patients compared to healthy controls.

Some studies on serum and bronchoalveolar lavage fluid (BALF) have been previously performed to find the differentially expressed proteins in IPF patients^16–19^. Out of these seven proteins found significant by volcano plot criteria, putting them in context of published literature revealed that CPB2 and HPR have been previously detected to be reduced in IPF patients’^16^. Five other proteins found significant by volcano plot are novel to our study. CPB2 in the said study^16^ was found to have 1.24, 1.87 and 2.14 times higher levels in serum of controls compared to IPF patients in three different comparisons compared to FC of 2.45 in our study. Directionality and magnitude of change was comparable to our study and the slight difference could be related to the use of serum vs plasma. HPR, although found to be different (FC 2.65–4.2 CTRL/IPF) in the same study was not proposed as a potential specific biomarker. However, we found it to be the best performing biomarker by a number of statistical techniques. The other serum study^17^ using iTRAQ found 97 differentially expressed proteins and four were validated which did not include our panel of 7 potential biomarker proteins. This study did not provide enough information to evaluate our proteins in comparison to their dataset. Additionally, two studies have been performed on BALF of IPF patients^18,19^, one of which compared familial vs sporadic cases of IPF^18^. Among the proteins quantitatively analyzed by this study ceruloplasmin had a fold change of 5.6 between familial vs sporadic IPF patients. Our study had a CV of 27.4% for this protein in the IPF group suggesting the discrepancies might be caused by two-dimensional electrophoresis vs shotgun proteomics comparison. Similarly other BALF study was also done with 2-DE based proteomics and did not have proteins common with our study’s differentially expressed proteins. We analyzed a recent large plasma proteomic dataset published by Bruederer *et al*.^20^ the inter-individual CV, for healthy individuals followed for weight loss and maintenance, was found to be 38%. Specifically for HPR the CV was 36.1% (1466 plasma samples from 433 individuals) and fold changes between baseline, 8 weeks of weight loss and 10 months of weight maintenance were 0.97 to 1.14. This further shows that on large population scale, having fold changes of 2 or more and CV of 36% with tightly controlled expression levels in larger cohort healthy population make HPR an attractive biomarker candidate for IPF. Despite lack of complete comparability due to different technical, instrument and cohort differences, it can be concluded that HPR in healthy individuals shows tight distribution. For the same reason our results need to be validated in larger international cohorts to establish the promise of HPR as a biomarker of IPF.

The top proteins significant in PLS-DA modeling can be divided into roughly three categories namely complement proteins, oxidation related proteins and protease inhibitors. Complement system has been suggested to play a role in development and progression of IPF which was inferred from early studies detecting complement-activating immune complexes and fragments of activated complement proteins in serum and bronchoalveolar lavage fluid of patients with pulmonary fibrosis^21–24^. The common gain-of-function MUC5B promoter variant (rs35705950) is a strong risk factor for development of pulmonary fibrosis^25^. Both complement system and MUC5B are implicated in host defense and this MUC5B variant is associated with higher C3 gene expression in lung tissue further indicating role of complement system in pulmonary fibrosis progression^25^. In our study, C3 was lower in IPF patients than healthy controls, which points towards consumption of this protein in lung tissues in patients with IPF. Lower C3 levels in IPF patients blood compared to healthy controls has been previously reported^26^ supporting our findings. C1q, C1s, C2, C4B, C5, C6 and C8 were also significantly lower in IPF patients compared to healthy controls. Another molecule which regulates alternative complement pathway, complement factor H (CFH), was found to be downregulated in IPF patients in our study. Plasma protease C1 inhibitor was also lower in IPF patients suggesting consumption in response to increased complement activation in IPF. This has been found in another study previously^26^. Whether, in response to putative higher complement activation in IPF lungs, host defense regulatory molecules (such as CFH) are consumed or they are inherently present in less amounts in IPF patients, that drives accelerated complement activation, remains to be established. Role of complement activation in IPF is an interesting and important area to study further.

Second class of proteins, which were present in significant proteins of PLS-DA, which could classify IPF from healthy controls was metal binding proteins/antioxidants which included hemopexin, ceruloplasmin and copper homeostasis protein cutC homolog. Hemopexin is an antioxidant transporter/enzyme which protects the body from oxidative damage of free heme^27^. Ceruloplasmin is a metal binding protein which transports and regulates the availability of copper in human plasma^28^. cutC is another copper transporter which transports the Cu to the target sites and limits its availability. Cu can be potentially toxic to the body and these proteins and enzymes protect our body from oxidative damage. Transition metals such as Cu regulate the redox reactions in our body and altered dynamics of their transporters/regulators can lead to disturbed metal ion homeostasis and redox balance. Elevated lipid peroxidation products, altered antioxidant enzyme levels and oxidized proteins have been reported in the epithelial lining fluid of the IPF patients^29–33^. It suggests oxidative stress plays important role in the pathogenesis of IPF^30^ and accordingly, redox-modulatory therapy for IPF has been envisaged^32^.

Top significant proteins by PLS-DA modeling in our study also included several protease inhibitors such as SERPINA3, SERPING1 and ITIH2 (Supplementary Table 2 in Supplementary dataset). These proteins were also significant by FDR corrected t-test p values (<0.05). SERPING1 is a regulator of the classical complement pathway and as discussed above, points towards dysregulation of complement system in IPF. SERPINA3 regulates fibrinolysis which is known to be altered in IPF^34^ and implicated in disease progression. Plasminogen was also found to be reduced in IPF patients compared to healthy controls in our study, which might indicate the consumption of plasmin and accordingly, elevated D-dimer concentration have been reported in IPF patients’ plasma^35^. Upon pathway analysis of all proteins quantified in our study complement and coagulation cascades were within the top 5 canonical pathways (B-H p-value <0.05). Taken together these observations indicate involvement of complement-coagulation system in development and/or progression of IPF. In network analysis of all proteins, proinflammatory cytokines, lipid metabolism and ERK1/2 pathways came up as hubs of the top network enriched with major modules being complement activation and coagulation cascade. Major lipid metabolism and transport pathways are known to be altered in lung diseases^36^. Although cholesterol is essential for lung function, excess of it was shown to interfere with normal surfactant function^37^. In line with these findings, agonists of cholesterol trafficking regulators, such as Liver X receptor reduces neutrophil recruitment and pro inflammatory responses^38^. Note that LXR/RXR activation was the top canonical pathway enriched in Ingenuity pathway analysis of all proteins. Moreover, in blood proteome of IPF patients ERK1/2 pathway and cytokine activity has been previously reported to be enriched^26^ lending support to our study.

One of the major aims of the current study was to find suitable predictive biomarkers which was performed by comparing FDR corrected p value of t-test (66 significant proteins) and proteins having VIP value of more than 1 in PLS-DA (30 proteins). Twenty four proteins were common to both the methods (Supplementary Table 2 in Supplementary dataset). When the additional filter of fold change of more than 2 was employed, only HPR out of the 24 proteins remained significant. HPR was 2.2 times reduced in IPF patients. HPR, like haptoglobin is a hemoglobin binding protein which help protects from toxic effects of free heme on the body. In haptoglobin-null mouse model, hemolytic stress leads to kidney injury due to oxidative damage^39^. In haptoglobin and hemopexin double null mouse model, hemolytic stress causes inflammation of liver, cirrhosis and splenomegaly^40^. Subsequent kupffer cells activation leads to secretion of pro-inflammatory cytokines culminating in fibrosis^40^. Recall that hemopexin is also found to be significant by VIP in our study and is reduced in IPF patients. Our dataset hints toward a similar mechanism of events in IPF, activation of complement leading to hemolysis and impairment of antioxidant function leading to inflammation and eventual fibrosis. This needs to be established by future mechanistic studies. HPR as a biomarker makes biological sense in IPF patients as oxidative stress related injury has been proposed as one of the mechanisms contributing to IPF progression. The mechanism of action of pirfenidone is now fully known, but it has been shown to work, at least partially, by regulating intracellular antioxidant functions thereby reducing oxidative stress in pulmonary fibrosis^15^. In a study of proteomics of BALF from IPF patients compared to controls, oxidative stress was found to be as one fo the enriched pathways by systems biology approaches^19^. HPR has also been shown to separate bacterial pneumonia from non-bacterial pneumonia in a previous study^41^. One of the potential shortcomings of our study is non-inclusion of pneumonia patients which have been ruled out for IPF. It would be interesting and informative to measure HPR levels in other respiratory diseases of known causes. Further validation of HPR in larger independent cohorts is warranted in future studies. In Fig. 4, ROC curve of HPR can be seen together with the optimal cutoff at which 80% of sensitivity and selectivity could be achieved. Considering there are no known biomarkers in clinical practice, our study provides a single candidate found by overlap and statistical validation by two independent techniques. When we use the term biomarkers here, it means detection biomarkers which would help indicate to the clinician the need for further clinical, radiological and histological testing in patients where no known causes (drug reaction, hazardous material exposure, systemic autoimmune diseases etc.) are apparent. It can further be validated in future studies as not only detection but also monitoring biomarkers where our working hypothesis is that treatment would lead to restoration, either full or partial, of HPR levels in IPF patients bringing them closer to healthy persons.

Our pilot study establishes HPR as a candidate biomarker of IPF and highlights dysregulated pathways from plasma proteome. Of note, our study has some limitations including modest number of IPF samples but  it provides impetus for future studies to validate our results in more numerous cohorts for IPF patients and other interstitial lung diseases. In view of lack of highly specific antibodies against HPR, to the best of our knowledge, mass spectrometry based assays would be the way forward for future validation of candidate biomarkers.

In conclusion, proteomic signature of plasma can be used to identify candidate biomarkers of IPF which are significant by multiple statistical procedures. Some of these candidates, including C3 and SERPING3, are previously known to be dysregulated in blood proteome of IPF patients. LXR/RXR activation, complement activation and coagulation cascade were among the top pathways enriched in plasma proteome of IPF patients as revealed by pathway and network analysis of proteomics dataset. Our study reveals HPR as a candidate marker of IPF, which should be validated in future studies.

## Materials and Methods

### Patients, Ethics and study design

All IPF patients were recruited from FinnishIPF registry^42^, which is a national registry study. Healthy age-, sex- and smoking status matched control samples are from the hospital based Helsinki Biobank. The Ethics Committee of the Helsinki University Hospital, Helsinki, Finland (permit numbers 426–13–03–01–09 and HUS/359/2017), approved statements for the use of samples. The study was carried out according to relevant guidelines and all patients gave written informed consent to participate in the study.

### Plasma sample processing

The blood samples were collected in EDTA plasma tubes and they were centrifuged at 2000g for 10 minutes to isolate plasma at +4 °C. Samples were frozen at −80 °C until used for the study. Plasma samples were processed essentially as described previously^43–45^. Briefly, plasma samples were thawed and 10 µL was used for depleting TOP 12 abundant proteins according to manufacturer’s instructions (Pierce, Thermo Fisher). Top 12 proteins depleted plasma was used for total protein estimation using BCA assay (Pierce, Thermo Fisher). Equal amount of protein from each sample was dried in separate tubes and dissolved in 35 µL of 50 mM Tris containing 6 M Urea (pH 7.8). After vortexing, dithiothreitol (DTT) was added to a final concentration of 10 mM and shaken for 1 hour at RT. Subsequently, 40 mM iodoacetamide (IAA, final concentration) was added to the tubes and shaken for 1 hour. Further, 40 mM DTT (final concentration) was added to the tubes to quench any remaining IAA for 1 hour on shaking. Protein solution was diluted with 1:10 with 50 mM Tris buffer (pH 7.8) and in a ratio of trypsin: total protein, 1:50, trypsin (Trypsin Gold, Promega) was added. The samples tubes were incubated at 37 °C for 18 hours without shaking. Next morning, samples were cleaned with C18 spin columns (Pierce, Thermo Fisher) according to manufacturer’s instructions. Samples were dried and reconstituted in 0.1% formic acid containing 50 fmol of Hi3 peptide mixtures (Waters, MA, USA) per 4 µL. Rationale for addition of Hi3 as a spiked standard is described below.

### UPLC-UDMS^E^

500 ng peptides were injected to nano Acquity UPLC (Ultra Performance Liquid Chromatography) ‐ system (Waters Corporation, MA, USA). TRIZAIC nanoTile 85 μm × 100 mm HSS‐T3u wTRAP was used to separate peptide by liquid chromatography before mass spectrometer. Samples were loaded, trapped and washed for two minutes with 8.0 μL/min with 1% B. The analytical gradient used is as follows: 0–1 min 1% B, at 2 min 5% B, at 65 min 30% B, at 78 min 50% B, at 80 min 85% B, at 83 min 85% B, at 84 min 1% B and at 90 min 1% B with 450 nL/min. Buffer A: 0.1% formic acid in water and Buffer B: 0.1% formic acid in acetonitrile.

Data independent acquisition using UDMS^E^ mode was performed with Synapt G2‐S HDMS (Waters Corporation, MA, USA). The collected data range was 100–2000 m/z, scan time one‐second and ion-mobility spectroscopy (IMS) wave velocity was fixed at 650 m/s. Triplicate runs for each sample were acquired and further analysis was done with Progenesis QI for Proteomics – software (Nonlinear Dynamics, Newcastle, UK).

## Data analysis

Data analysis was performed as described previously^43–45^. Briefly, the raw files were imported to Progenesis QI for proteomics software (Nonlinear Dynamics, Newcastle, UK). Ion, 785.8426 m/z, corresponding to doubly charged Glu1‐Fibrinopeptide B was used for lock mass correction. Default parameters were used for peak picking and chromatographic peak alignment. The software facilitated the peptide identification with Protein Lynx Global Server and label‐free quantification was according to Silva *et al*.^46^. The peptide identification was done against Uniprot human FASTA sequences (UniprotKB Release 2015_09, 20,205 sequence entries) with (CLPB_ECOLI (P63285)), ClpB protein sequence (Hi3 peptide mixture) inserted for label‐free quantification. Carbamidomethylation of cysteine as a fixed modification and oxidation of methionine as variable modification were used for database searching. Trypsin was used as digesting agent and two missed cleavage was allowed. Fragment and peptide error tolerances were set to auto and FDR to less than 1%. One or more ion fragments per peptide, three or more fragments per protein and one or more peptides per protein were required for ion matching. The identified proteins were grouped together according to parsimony principle and also peptides unique to the protein are reported. Top 12 abundant proteins depleted were removed from further analysis.

### Statistics

Scaling of the data, Hierarchical clustering, partial least square-discriminant analysis (PLS-DA) and receiver operating curve (ROC) analysis was performed by R-program based server MetaboAnalyst^47,48^. Individual normalized relative peptide intensities were used for pareto scaling of the data which were used for PLS-DA with all proteins. For PLS-DA, 10 fold cross validation was employed and permutation testing with 100 permutations was also performed. The differential expression analysis was done in four distinct steps. Briefly, 1. t-test was performed on the Control vs IPF group individual normalized abundances values and 0.05 was taken as threshold for considering significantly different proteins. FDR corrected p-values were calculated according to the method of Benjamini-Hochberg correction of raw p-values. 2. These FDR corrected p value significant proteins were further filtered to include only proteins which differed between the groups by fold change of 2 or above to give us a list of proteins. 3. Partial least square-discriminant (PLS-DA) analysis was performed on all proteins quantified in our study and variable influence on projections (VIP) values of 1 or more were considered significant. 4. Proteins significant in step 2 (FDR corrected p-value <0.05, FC of 2 or more) and step 3 (VIP values of ≥1) were compared and overlapping proteins were considered significantly differentially expressed. Data were analyzed through the use of IPA^49^ (QIAGEN Inc., https://www.qiagenbioinformatics.com/products/ingenuitypathway-analysis).

## Supplementary information

Supplementary Figures

Supplementary Tables

## Data Availability

The mass spectrometry proteomics data have been deposited to the ProteomeXchange Consortium via the PRIDE^50^ partner repository with the dataset identifier PXD010965 and 10.6019/PXD010965.
