# Supplementary material for: Label-free plasma proteomics identifies haptoglobin-related protein as candidate marker of idiopathic pulmonary fibrosis and dysregulation of complement and oxidative pathways
Source: Sci Rep. 2020 May 8;10:7787. doi: 10.1038/s41598-020-64759-x (PMC7211010; doi:10.1038/s41598-020-64759-x)
Supplement: Supplementary file 1 — Supplementary Figures [file 41598_2020_64759_MOESM1_ESM.pdf]

Label-free plasma proteomics identifies haptoglobin-related protein as candidate marker of idiopathic pulmonary fibrosis and dysregulation of complement and oxidative pathways

Mayank Saraswat, Sakari Joenväärä, Tiialotta Tohmola, Eva Sutinen, Ville Vartiainen, Katri Koli, Marjukka Myllärniemi, Risto Renkonen

Supplementary Figures

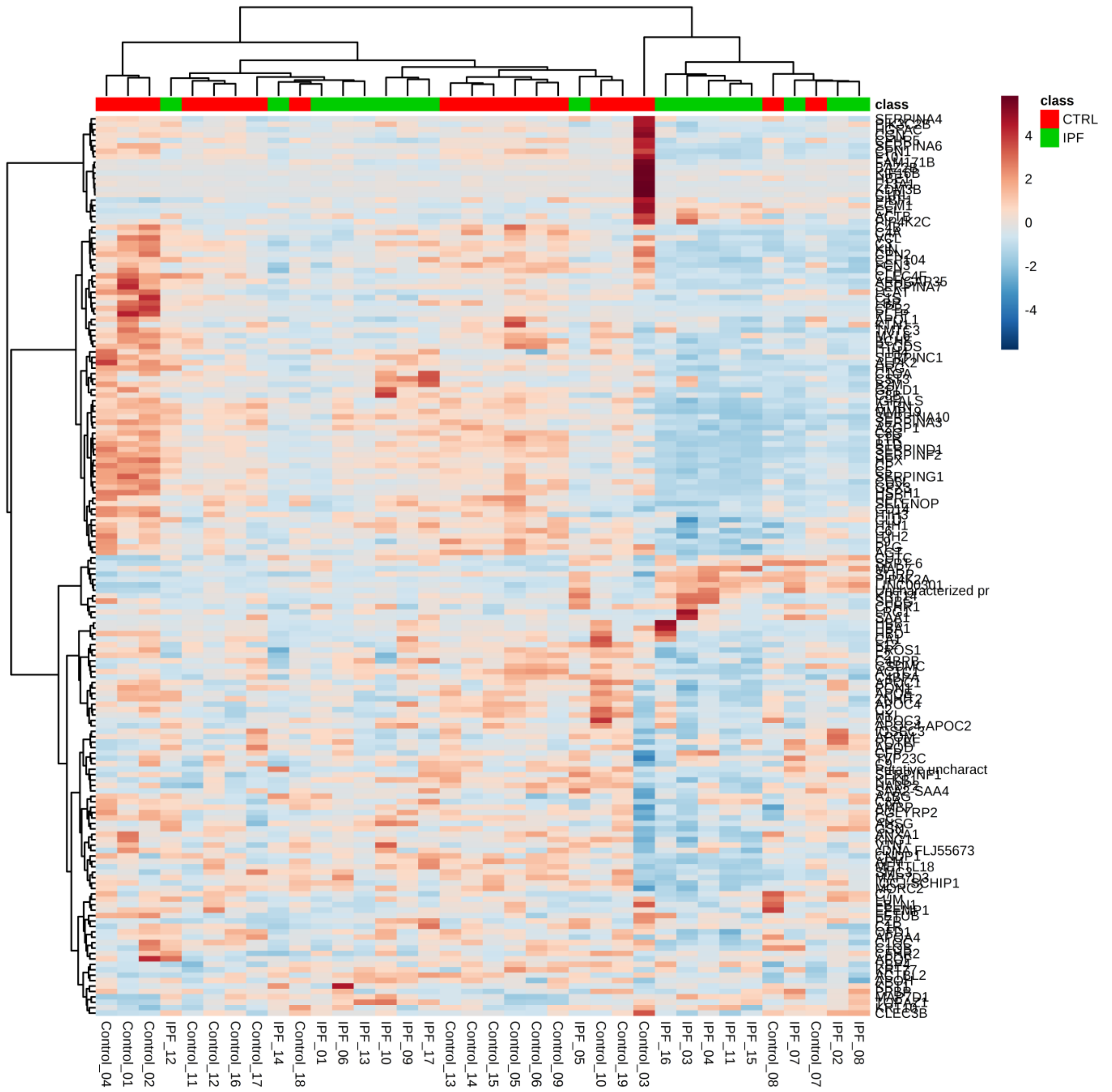

Supplementary Figure 1: Hierarchical clustering was performed for Controls and Idiopathic pulmonary fibrosis (IPF) comparison considering all proteins quantified in current study. Metaboanalyst 4.0 was used for performing the clustering. Horizontal axis denotes all the samples analyzed in the study and vertical axis denotes Uniprot accessions for the proteins. On top of the heatmap are controls samples in green squares and IPF samples (Cases) in red squares. Dendrogram for samples is shown on top of the heatmap and proteins' dendrogram on left side of the heatmap. Dark blue to dark red colour gradient denotes lower to higher expression.

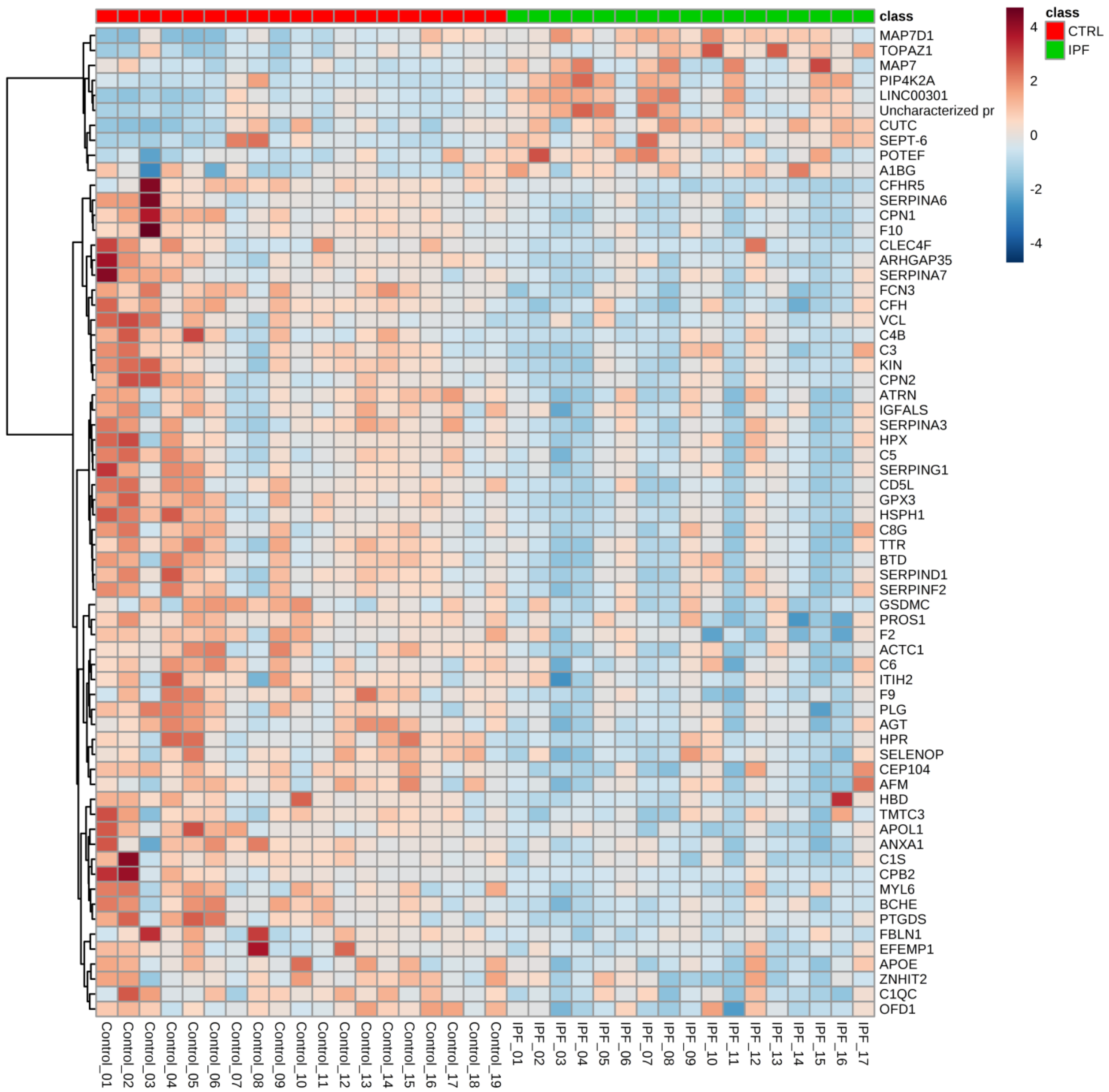

Supplementary Figure 2: Hierarchical clustering was performed by forcing the samples in each group to be organized together according to their group (Case=IPF, Controls=Healthy individuals) utilizing 66 differentially expressed proteins between the two groups (FDR corrected p-value <0.05). It caused the contrast in the protein expression between thee groups to be observed more clearly. Metaboanalyst 4.0 was used for performing the clustering. Here, 10

proteins increased in IPF patients and 56 in controls could be clearly seen to have a contrasting pattern.

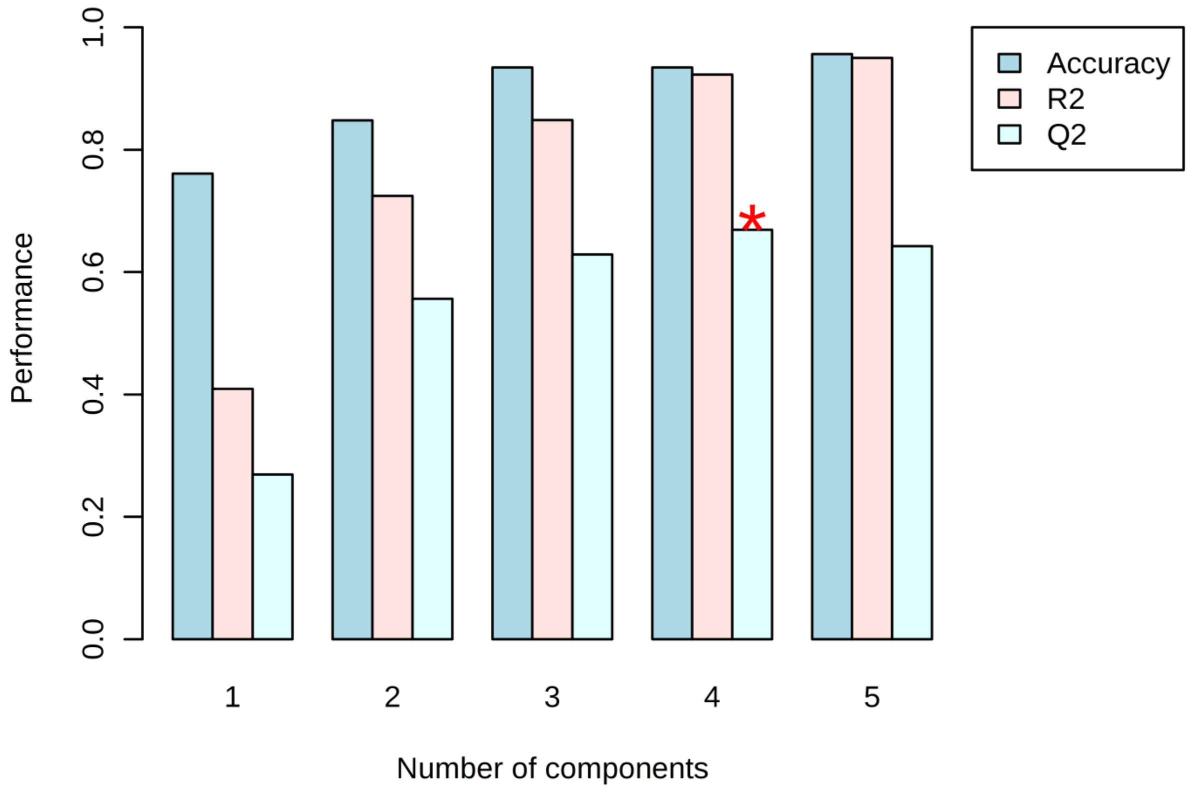

Supplementary Figure 3: Ten-fold cross validation was employed to test the fit (R^2), accuracy and predictive ability (Q^2) of the partial least square-discriminant analysis (PLS-DA) modeling performed on all the proteins quantified in the current study. Metaboanalyst 4.0 was used for performing the modeling. Five components were allowed but four components already gave the best output parameters.
